# The impact of the social isolation in elderly Brazilian mental health (anxiety and depression) during the COVID-19 pandemic

**DOI:** 10.3389/fpsyt.2022.888234

**Published:** 2022-09-08

**Authors:** Isabella Louise Morais de Sousa, Rodrigo Silveira, Mônica Yuri Takito, Adenilson Leão Pereira, Dalberto Lucianelli-Júnior, Giselle Sousa Carmona, Ana Paula do Vale Viegas, Francisco Bruno Teixeira, Ozélia Sousa Santos, Fernanda Nogueira Valentin

**Affiliations:** ^1^Faculty of Medicine, Federal University of Pará, Altamira, Brazil; ^2^Campus 'University City Armando de Salles Oliveira (CUASO)', University of São Paulo, São Paulo, Brazil; ^3^Graduate Program in Biodiversity and Conservation (PPGBC), Altamira, Brazil; ^4^Faculty of Medicine, Federal University of Piauí, Teresina, Brazil; ^5^Faculty of Medicine, State University of Pará, Belém, Brazil

**Keywords:** COVID-19, social isolation, elderly, mental health, anxiety, depression

## Abstract

The impact of social isolation in the pandemic context on elderly Brazilian mental health is little known, especially about the occurrence of depressive symptoms. In this study, we evaluated elderly people undergoing social isolation in order to identify factors associated with depression and which of these are more important to characterize elderly Brazilians with depression. In a cross-sectional, exploratory, and analytical study of a quantitative nature, the mental profile of elderly individuals subjected to social isolation during the COVID-19 pandemic period was used. A total of 450 participants was divided into normal and depressive groups, and a form covering sociodemographic data, opinions/perceptions about the pandemic, and a Reduced Geriatric Depression Scale was used to assess participants' mental health. To assess the statistical significance between the variables, chi-square test was applied, considering the *p*-value <0.05. The effect size was analyzed to identify the magnitude of the difference between groups. To identify the most important characteristics to define the groups Multilayer Perceptron algorithm were applied. We found that elderly people with a depressive profile are (in Multilayer Perceptron rank order) (1) showing signs of anxiety during the COVID-19 pandemic, (2) of low education, (3) being divorced, (4) having more than one mental disorder, (5) reading, watching, or listening to information about COVID-19, and (6) being previously diagnosed with depression. In conclusion, elderly Brazilians in social isolation tend to develop depressive disorders during quarantine. Thus, we can consider that the pandemic requires effective and safe gerontological care and monitoring, especially with regard to mental health.

## Introduction

The emergence and rapid increase in the number of cases of COVID-19, an infectious disease caused by the new coronavirus, which in most cases can lead the patient to the severe acute respiratory syndrome, presents complex challenges for health, economy, and society. COVID-19 is currently a public health emergency of international concern, as declared on 30 January 2020 by the World Health Organization. In early July of 2022, there were more than 552 million confirmed cases of COVID-19 worldwide and more than 6.34 million deaths (1).

The first confirmed case of COVID-19 in Brazil was announced on 26 February 2020. Currently, the number of cases exceeds 28 million and more than 670,000 victims, making Brazil the third country with more cases and is the second deaths by COVID-19 in the world (2).

The COVID-19 pandemic has been compared to catastrophic events such as earthquakes, tsunamis, conflicts, and wars. However, unlike these cases, the pandemic was and is still something unusual and obscure for world society, because until some time ago, it was not known what was ahead, and the possibility of contagion by the virus was everywhere and is still a threat (3). In addition, the excess of information transmitted and still may generate panic, favoring situations of stress and fear. Studies show that these factors can trigger traumatic stress, which may manifest itself in the main models of post-traumatic stress disorder (4, 5), which may have even more catastrophic impacts on vulnerable groups such as the elderly people (6, 7).

In the beginning of the confrontation of the COVID-19 pandemic, in 2020, Brazil adopted many public health measures, such as quarantine and mandatory social isolation, suspensions from school and non-essential services, in order to mitigate the risks and impact of the disease on the population. A study carried out in Hong Kong showed that sudden changes in daily life are risk factors that can substantially affect mental health, and this fact can be brought to the Brazilian context (8).

The elderly are more vulnerable to COVID-19 because they have a higher risk of developing the most severe form of the disease, especially those with preexisting comorbidities, such as heart, hypertension, diabetes, kidney, lung, cancer, and immunosuppression diseases (9). In Brazil, the mortality rate in 2020 among people with aged ≥80 years was higher (14.8% died), when compared to the elderly aged 70–79 years (8% died) and 60–69 years (8.8% died), in other words, a rate of 3.82 times higher than the general average, reinforcing the concerns regarding the elderly population (10). However, after the start of vaccination for the elderly in January 2021, these numbers have been reduced (11). Orellana and collaborators in 2022 (12) observed changes in the pattern of hospitalizations and deaths from COVID-19 after substantial vaccination of the elderly in Manaus, Amazonas, and Brazil. According to him, there was an overall reduction of approximately 62% in hospitalization and death rates, especially in the elderly aged 60–69 years.

Social distancing and isolation are among the recommended guidelines for the safety of the elderly during the pandemic. However, social isolation is a major danger to the health and wellbeing of the elderly as it is associated with an increased mortality risk and is linked to worsening mental health (13). The incidence and prevalence of the depressive disorder in the elderly population is high globally, and although it affects both sexes, the incidence is higher in women (14). Recently, Santini, Jose (15), observed that social disconnection exposes the elderly to a high risk of depression. In addition, it is believed that the health risks associated with the social isolation and loneliness are equivalent to the prejudicial effects caused by smoking and obesity (16).

The causes of depression can be genetic, brain biochemistry, or vital events. Events that cause stress and anxiety, also called vital events, are mostly triggering factors for depressive episodes, especially in those who already have a genetic predisposition to the development of the disorder. The imbalance of neurotransmitters such as serotonin, dopamine, and noradrenaline responsible for controlling appetite, mood, and motor activity are also closely associated with depression (17).

The situations of daily life trigger different reactions in individuals, among which depressive symptoms are present. In these situations, individuals demonstrate general or non-specific responses of a physiological and psychological nature of the body to a stressor or external and internal threats (18, 19).

The causes and symptoms that trigger the depressive disorder are well characterized; however, in elderly individuals, these symptoms are more difficult to diagnose and, consequently, to treat. Therefore, the main difficulty in the treatment of this clinical condition is the correct diagnosis, which is partly associated with the fact that many elderly people do not accept their depressive clinical condition and do not seek adequate psychiatric treatment (20). In this scenario, the context of the COVID-19 global pandemic can make this situation more aggravating, since the fear of the unknown can lead to depression, and social isolation measures limit people's daily activities, especially of the elderly (21). Therefore, due to the pandemic conditions to which the elderly are being subjected, the development or worsening of depressive conditions is expected, since these disorders are closely related to social isolation, affecting physical and mental health and aggravating underlying diseases (22).

The Brazilian population has a cultural and religious plurality that is very subjective (23, 24), and it is possible that it does not behave in the same way in relation to other population groups. In this context, the use of machine learning can be useful to create robust models that can provide more accurate data for this population.

Although there are previous works based on bibliographic reviews in Brazil (25–28), and some cross-sectional studies on mental health of the elderly in the pandemic in other countries such as China, Spain, and Italy (8, 29–33), in Brazil, cross-sectional studies have not yet been found, nor combined with k-means cluster analysis (an unsupervised machine learning algorithm) that explored the association of COVID-19 impacts and physical isolation on the mental health of elderly Brazilians, especially in terms of depression levels.

In this study, we aimed to identify whether there are distinct groups in the elderly population (with and without depression). We also analyzed the main characteristics of elderly Brazilian people with and without depression in the period of social isolation and we identified which of these characteristics are more important to characterize Brazilian elderly people with depression. Thus, based on the literature cited, it is believed that elderly Brazilians may develop or worsen depressive symptoms during the COVID-19 pandemic due to social isolation.

## Materials and methods

### Participants

The study included 450 male and female subjects, over 60 years of age (67.2 ± 6.7 years), representing all Brazilian states. The form was in Portuguese and was available online from 26 June to 8 September 2020, through social networks and e-mail.

Data collection was performed after approval of the research project by the Ethics Committee of the Institute of Health Sciences of the Federal University of Pará (CAAE number: 32893620.8.0000.0018). All participants who agreed to participate in the research signed the Informed Consent Form.

In this study, only people who lived in Brazil at the time of data collection were included. The questionnaires were distributed mostly by e-mail to universities, institutes, and personal e-mails of project participants. In addition, another part of the participants, the application of the form, was carried out through the whatsapp application and/or telephone call. The elderly who could not answer the form alone were helped by someone close (family member, friends, or project participants) to whom the questions were dictated and the respective alternatives were answered verbally. Participants unable to answer verbally and/or provide decisions regarding the alternatives to the questions by cognitive or psychiatric disability were excluded. In addition, for all participants who filled it more than once, only the first participation was maintained, excluding the remaining.

To ensure better quality of the data obtained, a pilot study was conducted before starting the official form dissemination with a dataset of 100 participants (not counted in the sample) to evaluate the dissemination strategy, responses obtained, and the quality of the anchoring questions.

For the sample calculation, the G^*^ Power 3.0.10 software was used to simulate all the analyses performed. The sample size was determined by the analysis that estimated the largest number of participants, being a Chi-square test with up to 6 degrees of freedom, assuming an intermediate effect size, a significance of *p* < 0.05 and a statistical power of 95%, estimating a minimum sample of *n* = 232. However, to ensure better representativeness of the Brazilian population, this minimum sample size was estimated to be increased by 90%. Thus, based on cultural plurality rooted in the great social and regional diversity in the set of 27 Brazilian states (34), the estimated minimum sample size increased by 186 (~80%) with an additional 22 (~10%) for possible sample loss, totaling a minimum sample size of *n* = 440.

The online form was structured with multiple choice questions and covering general demographic data such as age, gender, race, marital status, religion, having children, education, city and previous diagnosis of mental disorder. The questions on the opinions and perceptions of the elderly regarding the COVID-19 pandemic were as follows: (a) If the participants claim to know what the pandemic and COVID-19 are?; (b) What are the main ways to obtain information about the pandemic?; (c) How much time do you spend getting this information?; (d) Do you know what social isolation is?; (e) Do you agree with the imposed social isolation?; (f) How do you feel about the whole pandemic scenario?; and (g) Who are they with passing the period of social isolation?

### Mental health measurements

To assess anxiety, the Brazilian version of the Geriatric Anxiety Inventory (GAI) with 20 objective questions was applied (34). The GAI is characterized by being a self-applicable instrument with dichotomous responses (agree/disagree) (35). The instrument has a cutoff score between 10/11 (non-case/case), where a score of 0–10 indicates no anxiety, 11–15 indicates mild or moderate anxiety, and 16–20 indicates severe anxiety. In this study, only the absence (score 0–10) or presence (score 11–20) of anxiety was considered.

To assess depression, the Brazilian version of the reduced Geriatric Depression Scale with 15 objective questions was applied (36). Its score ranges from 0 to 15 points, being divided into three categories. A score of 0–5 is considered normal, 6–10 mild depressive symptoms, and 11–15 severe depressive symptoms. We only considered the absence (score 0–5) or presence (score 6–15) of depression.

### Bias

To avoid possible interpretation errors and potential sources of bias, a pilot study was conducted, which served to improve the form questions.

### Data analysis

Continuous data were presented as the median and interquartile range, while categorical data as percentages. To analyze the significance between the proportions of the sample with and without depressive disorder, 95% confidence intervals were observed. To analyze the associations between the groups with and without depression and the different categorical variables, Pearson's chi-square test was applied. Correction by Fisher's exact test was applied when in any contingency table there was *n* < 6 in any cell. For all tests, the statistical significance adopted was *p*-value <0.05. In contingency tables >2 x 2 with statistical significance, adjusted residuals >2 were analyzed to identify which categories influenced the *p*-value <0.05.

To analyze the magnitude of differences between groups, effect sizes were observed using *Φ* (ϕ) in 2 x 2 tables, assuming “no effect” for ϕ < 0.10, “small effect” for ϕ < 0.30, “moderate effect” for ϕ < 0.50 and “large effect” for higher values. In >2 x 2 tables, the sizes were observed by Cramer's V, whose interpretations of null, small, moderate, and large effects were performed considering the variations according to an increase in degrees of freedom (37, 38).

To assess the characteristics that most influence the classification of the participants as depressive or non-depressive, the Multilayer Perceptron algorithm was used (*p*-value <0.05). This supervised machine learning algorithm, through an artificial neural network, identifies non-linear patterns among different variables in a dataset and, in response, provides a prediction of some predetermined variable of interest. When executed, this learning algorithm perform through the following steps: (1) the weights are initialized; (2) the flow and analysis of information flows through the input, hidden, and output layers; (3) error rate in output layer predictions is calculated and weights are adjusted; and (4) all previous steps are repeated until the error rate becomes as low as possible (39).

Quantitative variables were rescheduled at intervals between 0 and 1. The samples were randomly divided into two datasets, where 70% of the samples were used for training the algorithm and 30% for the test. For training and optimization, Minibatch and Descending Gradient methods were selected, respectively. Because Multilayer Perceptron can give different results each time it is run due to randomization of dataset partitions for cross validation and initialization of weights, the algorithm was run three times. The trial chosen was the one with the lowest mean value of cross-entropy error ([training error + test error]/2). Therefore, the chosen attempt was the second.

The ability of the predictors to determine the artificial neural network was tested by using sensitivity analysis, combining the training and test samples. In addition, a table that shows the degree of importance of each predictor was created. Data analyses were processed using the SPSS v.23.0 software.

## Results

**Figure 1:**
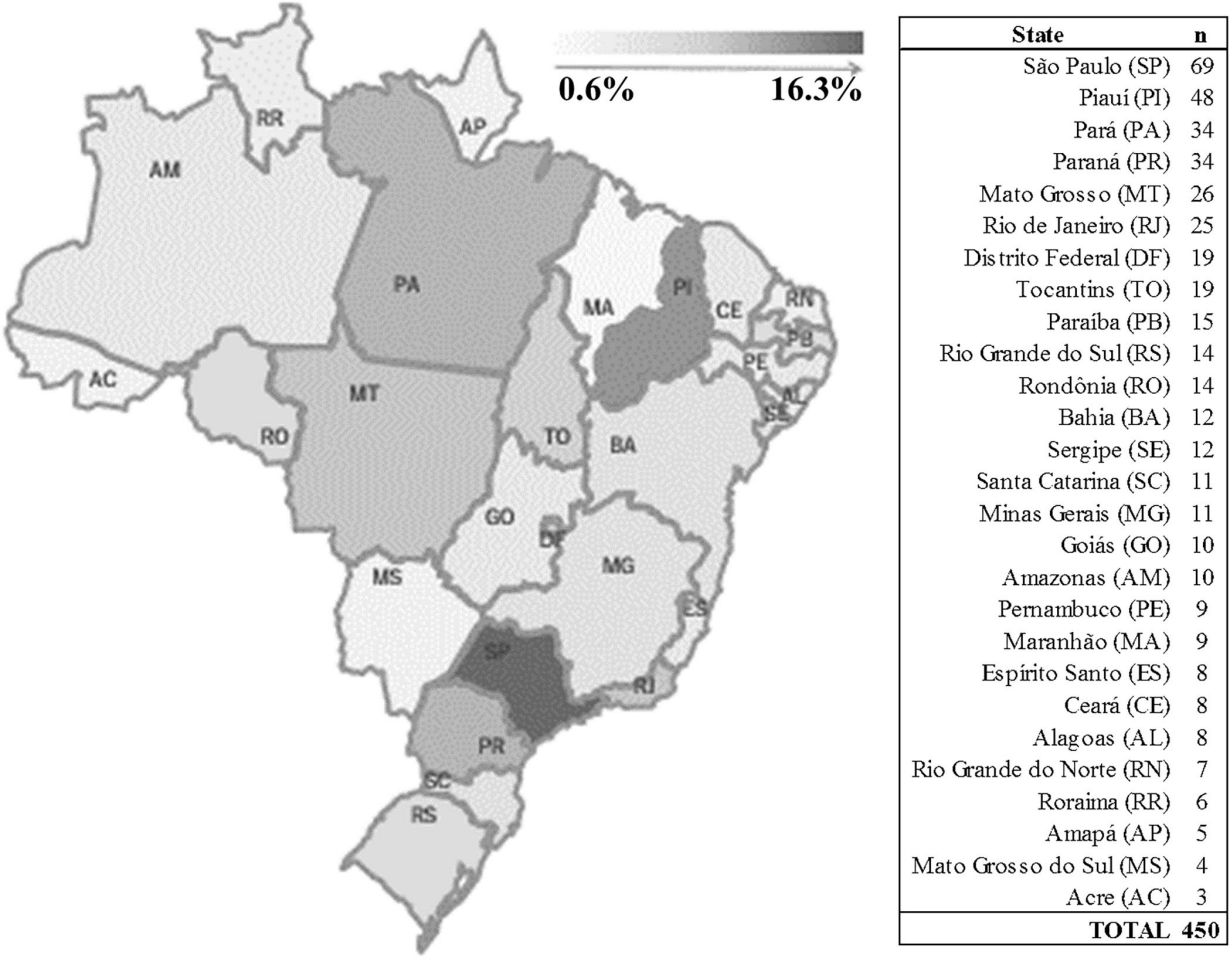
Sample divided by Brazilian states.

The sample distribution (*n* = 450) across Brazilian states ranged from *n* = 3 in Acre to *n* = 69 in São Paulo (Figure 1). Of the 450 subjects, 31.1% showed depressive symptoms (IC: Normal = 64.6–73.2; IC: Depressive = 26.8–35.4).

The sociodemographic characteristics between the groups are detailed in Supplementary Table S1. Individuals with depressive symptoms are characterized by having a higher proportion of women (80.7%), divorced (23.6%), and with low education (32.9%) (*p* < 0.01). Regarding the religion of the elderly without depressive symptoms, there was a higher proportion of individuals without religion (14.2%), while among the elderly with depressive symptoms, there was a higher proportion of subjects who adhere to Afro-Brazilian religions (2.1%) (*p* < 0.05). There was no statistical difference between the depressive and normal groups regarding ethnicity and whether they had children.

Table 1 shows the participants' psychological responses and perceptions about the COVID-19 pandemic. The elderly people were divided into groups with and without depression. On declaring themselves to have a mental disorder, it was observed that the elderly people who claimed to be anxious and those who already had a diagnosis of depressive disorder were present in the group with depression (*p* < 0.001). On declaring themselves to have a mental disorder, it was observed that the elderly who claimed to be anxious and those who already had a diagnosis of depressive disorder were present in the depressive group (*p* < 0.001). There is a direct relationship between the number of mental diseases and the group that has depression, while people without any mental disorder are mostly present in the non-depressive group.

**Table 1:** Psychological responses and participants' perceptions about the COVID-19 pandemic.

	**Psychological responses and participants' perceptions of the COVID-19 pandemic**				
		**Depression (*n* = 140)**	**Normal (*n* = 310)**	**Effect size**	***P***
Diagnosed with some mental disorder	Anxiety	24.30%	8.10%	ϕ = 0.22^+^	<0.001^***^
	Depression	29.30%	9.00%	ϕ = 0.26^+^	<0.001^***^
	Bipolar affective disorder	1.40%	1.00%	ϕ = 0.20^+^	0.65
	ADHD	0.70%	1.30%	ϕ = 0.25^+^	1
	Panic syndrome	2.10%	0.60%	ϕ = 0.07^+^	0.18
Number of mental disorders	Any mental disorder	53.6%^a^	82.3%^a^	*v* = 0.31^++^	<0.001^***^
	Has one mental disorder diagnosed	35.0%^b^	15.5%^b^		
	Has two mental disorders diagnosed	11.4%^c^	2.3%^c^		
Classification of anxiety	Shows signs of severe anxiety during the COVID-19 pandemic	30.7%	1.6%	*v* = 0.54^+++^	<0.001^***^
	Shows signs of mild (leve) to moderate anxiety during the COVID-19 pandemic	25.0%	6.8%		
Understanding about COVID-19 pandemic	Declares not understand the world situation due to the COVID-19 pandemic	19.30%	9.00%	ϕ = 0.14^+^	<0.01^**^
	Declares not understand what is a pandemic and COVID-19	23.60%	11.90%	ϕ = 0.15^+^	<0.01^**^
Source of information about COVID-19	Declares usually reads. watches or listens to news related to COVID-19	87.90%	94.80%	ϕ = 0.12^+^	<0.01^**^
Information source most consulted for news about the COVID-19 pandemic	World Health Organization Guidelines	7.90%	6.80%	*v* = 0.11^++^	0.13
	Radio and Televison	62.90%	54.20%		
	Internet and magazines	20.70%	31.60%		
	Family	8.60%	7.40%		
Reasons for social isolation reported by participants	Prevent the spread of the virus	37.90%	42.90%	*v* = 0.14^++^	<0.05^*^
	He / she is in the risk group	10.70%	6.10%		
	For him / her not to be contaminated	49.30%	43.20%		
	Does not know the reason for the social isolation or did not know how to explain	2.1%^a^	7.7%^a^		

Different letters represent the categories that influenced the statistical significance (p < 0.05) between the groups, with the letter “a” corresponding to the highest adjusted-value residual (>2) and the subsequent letters characterizing smaller values, respectively.

+ small effect,

++ moderate effect,

+++ large effect.

* *p* < 0.05,

** *p* < 0.01,

*** *p* < 0.001.

Elderly with signs of severe (30.7%) and mild-to-moderate (25.0%) anxiety were predominant in depressive group (*p* < 0.001), having a large effect size (*p* < 0.001). Those who declared that they do not understand the situation that the world is going through, and who do not understand what a pandemic and COVID-19 is, most of them are present in the depressive group. Those who usually obtain information through reading, viewing, or listening to news about COVID-19 are present in the non-depressive group. There was no relevance among the sources of information used by the elderly to find out about the pandemic. As for the reason why the elderly person maintains social isolation, the elderly who declared not knowing or not understanding the reason for physical isolation predominated in the non-depressive group; this variable was the most influential (*p* < 0.05).

**Figure 2:**
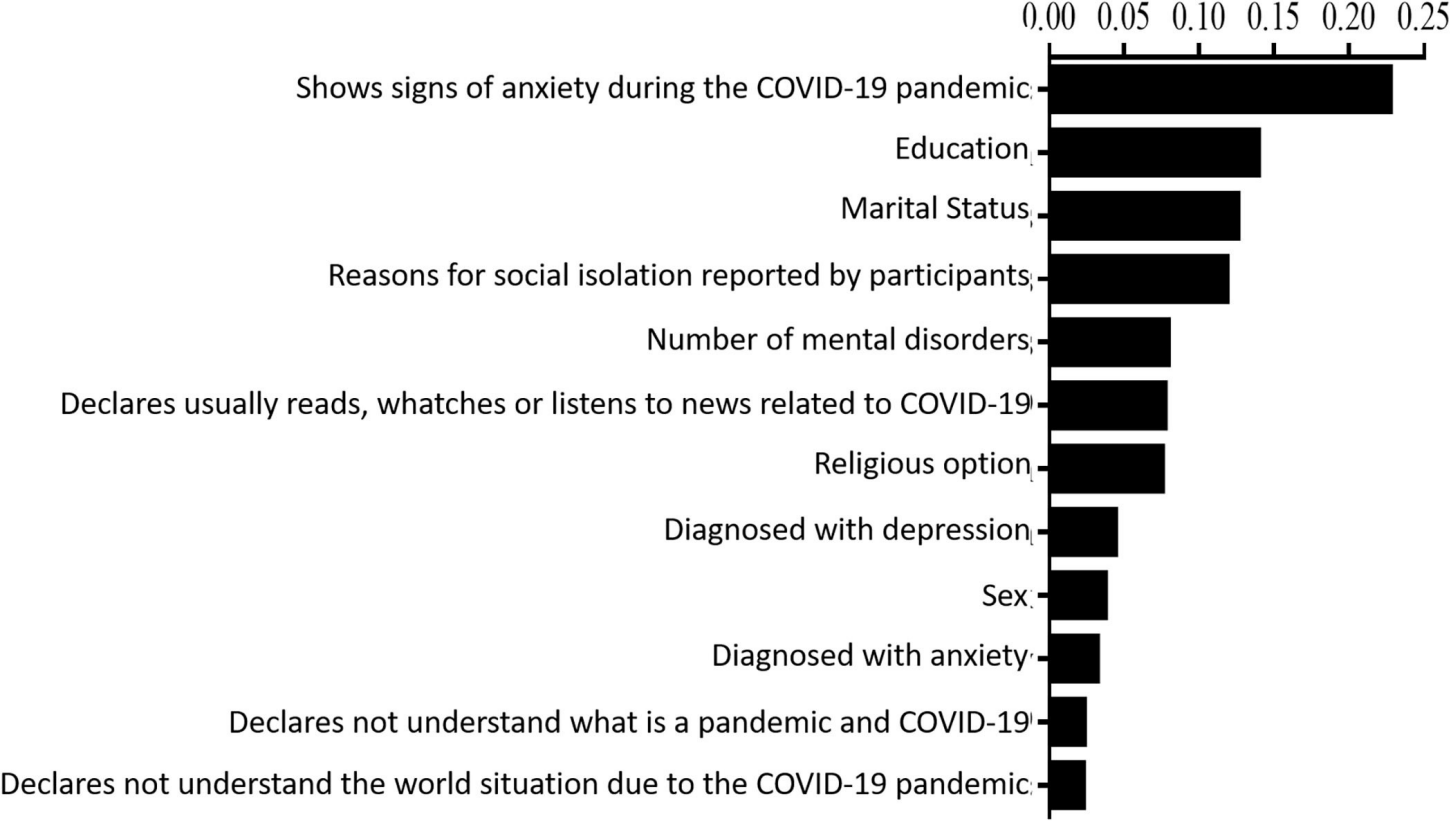
Importance of the variables for the characterization of the groups with and without depression. The figure shows the percentage importance of each variable that has statistical significance.

Of the characteristics with statistical value (*p* < 0.05), the most important to identify the groups is the presence or absence of anxiety symptoms, followed by education and civil status (Figure 2). In addition, the ranking showed that the importance of the other variables varies in a complex way among biological, psychological, and social factors.

## Discussion

The COVID-19 pandemic was and is still considered an acute stressor for the general population (40). In addition, studies have shown that this event generated emotional deregulation that culminates in high psychological distress, triggering anxious and depressive symptoms, especially for older age groups (41, 42). Such an event contributed to a large number of people developing and exacerbating neurological disorders, which are determined by individual factors that affect the way each patient deals with a traumatic event, such as the pandemic (43).

In this study, we evaluated and identified the characteristics of Brazilian elderly people with and without depression in social isolation during the COVID-19 pandemic period, and which of them are more appropriate to characterize the elderly in depressive conditions. In general, the elderly in depressive conditions are mostly diagnosed with anxiety, have low education, and are widowed or unmarried.

We observed a predominance of women in the depressive group. This fact may be linked to the fact that women tend to be more vulnerable when subjected to stress and when developing post-traumatic symptoms, as a consequence of the intense routine required by the demands of work, child care, and daily routines (44). Our results corroborate previous studies (45, 46) that found an association between the female sex and psychological distress increasing.

Studies have observed that during social isolation there has been an increase in the number of cases of domestic violence against women in Brazil, in part, as a result of the longer time spent with couples or spouses (47, 48). The rise in this type of violence was an important factor for the development of depressive symptoms in women (47, 48).

We found that marital status is also associated with depression levels. In fact, widowed or divorced elderly people have a higher risk of feeling lonely and depressed (49). The loss of the spouse can cause an increase in depressive symptoms, and the absence of a partner is among the factors that lead the elderly to a state of social and emotional loneliness, favoring the onset of depressive symptoms (50).

Regarding religious conviction, the elderly of Afro-descendant religions belonged to the depressive group, while the elderly belonging to the non-depressive group and more informed about the pandemic declared not to have a religion. Therefore, we emphasize that new studies considering religious conviction among depressed elderly people need to be conducted to better investigate, characterize and understand the impact of this variable on the mental health of elderly people.

The fact of having or not having children was not statistically significant in determining the groups with and without depression. Nóbrega et al. (51) observed that the presence of depression in elderly Brazilians was independent of the fact of having children. Oliveira et al. (52) observed that elderly people who do not live with their children have a higher risk of feeling depressed, probably due to the feeling of loneliness. We emphasize that there is no consensus whether this variable is a factor directly related to the presence of depression in elderly.

The second most important variable to characterize depressed elderly people was their low educational level. These results corroborate previous studies that report that this condition influences the onset of anxiety and depression symptoms during old age (53, 54). The educational level is directly related to the economic level and quality of life, factors that are determinant for the index of depressive symptoms (55). It is recognized that the educational level is directly related to the economic level and quality of life, factors that are determinant for the index of depressive symptoms (44). These combined characteristics provide a state of pessimism that may result in the inability to confront these situations (56). In addition, the inability to read and interpret texts combined with limited access to information can be an obstacle for the elderly to obtain a minimum level of knowledge about protective measures against the coronavirus and to update themselves on their reality. Thus, this group may develop more concerns and, consequently, become more prone to the development of depressive symptoms (56).

Regarding the fact of having depression and previous diagnosis of other mental illnesses, the most elderly people with depressive disorders claimed to have another type of psychiatric disorder, mainly anxiety. We also identified that the most influential variable in determining elderly people with depressive disorder is the previous diagnosis of anxiety, since 55.7% of the elderly reported having symptoms of anxiety during the COVID-19 pandemic. These results corroborate the results of studies carried out in other countries during the pandemic (29). Anxiety is considered a possible risk factor for the onset of depression, and the simultaneous occurrence of these two psychopathologies among the elderly is frequent (57).

Elderly people in the non-depressive group stood out in terms of obtaining information about the pandemic and COVID-19 when compared to the depressive group. We emphasize that the individual in depression may develop feelings and thoughts of pessimism, helplessness, deep sadness, apathy, lack of initiative, physical discontent, difficulty in organizing and fluidity of ideas, impaired cognitive judgment, among other symptoms (58). Thus, such factors can compromise the ability of an individual affected by depression to obtain information, especially when related to COVID-19.

Participants who declared not knowing or not understanding the reason for physical isolation were predominant in the non-depressive elderly group. This result may be a consequence of data collection since the data were collected at the beginning of the pandemic, when the rigor of preventive measures imposed on the elderly population was lower and this group had no discernment of the COVID-19 complications. Thus, they probably became more prone to social isolation and, consequently, did not develop depressive symptoms.

We consider that the use of the electronic form could be a limitation for this study, since it could induce subjectivity in the interpretation of questions by the participants. To minimize this bias and before starting the study, we applied a pilot form with the aim of evaluating and improving the quality of the questions, alternative answers and avoiding possible misinterpretations. As a result of the adjustments, the final form is easier and clearer for elderly understanding.

Another limitation of this study was the impossibility of selecting, through “selection criteria,” only elderly people with the ability to handle electronic devices. This fact may have restricted the number of people who could have participated in the study, and consequently, may have been a bias. However, many of the elderly participants had the help of family members with such skill during the completion of the form, which may have reduced this bias. Although the study included participants from all Brazilian states, the predominance of women among the participants may have interfered with gender representation and may be a bias in terms of Brazilian population representation.

This study is important because it evaluated elderly people from all Brazilian states, which allowed the identification of the main mental characteristics of Brazilian elderly people affected by the pandemic period, considering the ethnic, social, and cultural plurality of this population (59). In additon, in this study, it was possible to recruit a large number of the participants and it was the only one to characterize the profile of mental health and the prevalence of depression associated with the pandemic period in the Brazilian elderly population.

With the results obtained in the study, which made it possible to know the characteristics of the elderly who developed or worsened symptoms of anxiety and depression, therapeutic strategies aimed at groups that are more likely to be anxious and depressive can be devised. People with mental illness or who share the characteristics found in the research may be unable or unwilling to protect themselves against COVID-19 due to apathy, depression, paranoia, or other psychiatric symptoms. Therefore, early identification of these symptoms is of fundamental importance for the resolution of the condition of these patients (60).

## Conclusion

Overall, this study identified that for the sample of elderly people studied, the most important characteristics to identify the group with depression during the COVID-19 pandemic were (1) showing signs of anxiety during the COVID-19 pandemic; (2) of low education; (3) being divorced; (4) having more than one mental disorder; (5) reading, watching, or listening to information about COVID-19, and (6) being previously diagnosed with depression.

In conclusion, elderly Brazilians in social isolation tend to develop depressive disorders during quarantine. Having anxiety, low education, and marital status were the most important variables to characterize the depressive group. Thus, we can consider that the pandemic requires effective and safe gerontological care and monitoring, especially with regard to mental health.

## Data availability statement

The original contributions presented in the study are included in the article/Supplementary material, further inquiries can be directed to the corresponding author/s.

## Ethics statement

Data collection was performed after approval of the Research Project by the Ethics Committee of the Institute of Health Sciences of the Federal University of Pará (CAAE number: 32893620.8.0000.0018). The patients/participants provided their written informed consent to participate in this study.

## Author contributions

IS, OS, and FV: study conception and design. IS, RS, DL-J, AP, GC, AV, FT, OS, and FV: methodology and data collection. RS and MT: modeling and statistical analysis. IS, GC, AV, and FT: descriptive analysis. AP and DL-J: article editors. RS, MT, AP, DL-J, OS, and FV: scientific consultants and correction supervision. All authors contributed to the article and approved the submitted version.

## Funding

We are grateful to PROPESP/UFPA (PAPQ) for funding the article publication fee. This study was partially funded by the Institutional Program for Scientific Initiation Scholarships (PIBIC) of the Federal University of Pará (UFPA), contemplating a scholarship (PRO4538-2020).

## Conflict of interest

The authors declare that the research was conducted in the absence of any commercial or financial relationships that could be construed as a potential conflict of interest.

## Publisher's note

All claims expressed in this article are solely those of the authors and do not necessarily represent those of their affiliated organizations, or those of the publisher, the editors and the reviewers. Any product that may be evaluated in this article, or claim that may be made by its manufacturer, is not guaranteed or endorsed by the publisher.
