# Supplementary material for: The impact of the social isolation in elderly Brazilian mental health (anxiety and depression) during the COVID-19 pandemic
Source: Front Psychiatry. 2022 Sep 8;13:888234. doi: 10.3389/fpsyt.2022.888234 (PMC9493187; doi:10.3389/fpsyt.2022.888234)
Supplement: Supplementary file 1 [file Table_1.DOCX]

**Table S1.** Participants’ sociodemographic characteristics

|  | **Sample characteristics** | | | | |
| --- | --- | --- | --- | --- | --- |
|  |  | **Depression**  **(n =140)** | **Normal**  **(n =310)** | **Effect Size** | ***p*** |
|  | Age (Years) | 66.5 (62 - 72) | 66.0 (62 - 70) |  | 0.22 |
|  | Sex | F = 80.7%^a^  M = 19.3%^a^ | F = 66.5%^a^  M = 33.5^a^ | *φ* = 0,14^+^ | < 0.01** |
| **Ethnicity** | Black | 7.10% | 5.50% | *v* = 0,56^+^ | 0.796 |
|  | Brown | 34.30% | 31.60% |  |  |
|  | Indigenous | 0.70% | 0.30% |  |  |
|  | White | 56.40% | 60.60% |  |  |
|  | Yellow | 1.40% | 1.90% |  |  |
| **Marital Status** | Single | 8.60% | 14.20% | *v* = 0,18^++^ | < 0.01** |
|  | Married or in a stable relationship | 45.7%^b^ | 57.4%^b^ |  |  |
|  | Divorced | 23.6%^a^ | 13.2%^a^ |  |  |
|  | Widowed | 22.10% | 15.20% |  |  |
| **Have children** |  | 92.10% | 89.00% | *φ* = 0.05^+^ | 0.31 |
| **Religious option** | No religion | 7.1%^b^ | 14.2%^b^ | *v* = 0.18^++^ | < 0.05* |
|  | Christian (Catholic) | 55.00% | 55.50% |  |  |
|  | Christian (Protestant) | 25.00% | 17.70% |  |  |
|  | Afro-Brazilian religions (Umbanda or Candomblé) | 2.1%^a^ | 0.0%^a^ |  |  |
|  | Judaism | 0.00% | 0.60% |  |  |
|  | Spiritist Kardecism | 10.00% | 11.60% |  |  |
|  | Not specified | 0,70% | 0,30% |  |  |
| **Education** | Elementary School or lower | 32.9%^b^ | 20.0%^b^ | *v* = 0.24^++^ | < 0.001*** |
|  | High School | 21.4%^c^ | 12.9%^c^ |  |  |
|  | Higher Education | 27.90% | 26,80% |  |  |
|  | Post Graduation | 17.9%^a^ | 40.3%^a^ |  |  |

Different letters represent the categories that influenced the statistical significance (p<0.05) between the groups, with the letter “a” corresponding to the highest adjusted residual value (>2) and the subsequent letters characterizing lower values, respectively.
